# Towards a global One Health index: a potential assessment tool for One Health performance

**DOI:** 10.1186/s40249-022-00979-9

**Published:** 2022-05-22

**Authors:** Xiao-Xi Zhang, Jing-Shu Liu, Le-Fei Han, Shang Xia, Shi-Zhu Li, Odel Y. Li, Kokouvi Kassegne, Min Li, Kun Yin, Qin-Qin Hu, Le-Shan Xiu, Yong-Zhang Zhu, Liang-Yu Huang, Xiang-Cheng Wang, Yi Zhang, Han-Qing Zhao, Jing-Xian Yin, Tian-Ge Jiang, Qin Li, Si-Wei Fei, Si-Yu Gu, Fu-Min Chen, Nan Zhou, Zi-Le Cheng, Yi Xie, Hui-Min Li, Jin Chen, Zhao-Yu Guo, Jia-Xin Feng, Lin Ai, Jing-Bo Xue, Qian Ye, Liz Grant, Jun-Xia Song, Geoff Simm, Jürg Utzinger, Xiao-Kui Guo, Xiao-Nong Zhou

**Affiliations:** 1 School of Global Health, Chinese Center for Tropical Diseases Research, Shanghai Jiao Tong University School of Medicine, Shanghai, People’s Republic of China; 2 One Health Center, Shanghai Jiao Tong University-The University of Edinburgh, Shanghai, People’s Republic of China; 3 National Institute of Parasitic Diseases at Chinese Center for Disease Control and Prevention (Chinese Center for Tropical Diseases Research), NHC Key Laboratory of Parasite and Vector Biology, WHO Collaborating Centre for Tropical Diseases, Shanghai, People’s Republic of China; 4 Shanghai Legislative Research Institute, Shanghai, People’s Republic of China; 5 Zhuhai Branch, ESPRE, Beijing Normal University Zhuhai Campus, Zhuhai, Guangdong People’s Republic of China; 6 Global Health Academy, The University of Edinburgh, Edinburgh, UK; 7 Food and Agriculture Organization of the United Nations, Rome, Italy; 8 Global Academy of Agriculture and Food Systems, The University of Edinburgh, Edinburgh, UK; 9 Swiss Tropical and Public Health Institute, Allschwil, Switzerland; 10 University of Basel, Basel, Switzerland

**Keywords:** Antimicrobial resistance, Cell-like framework, Climate change, Food security, Global One Health index (GOHI), Global performance assessment, Governance, Zoonotic diseases

## Abstract

**Background:**

A One Health approach has been increasingly mainstreamed by the international community, as it provides for holistic thinking in recognizing the close links and inter-dependence of the health of humans, animals and the environment. However, the dearth of real-world evidence has hampered application of a One Health approach in shaping policies and practice. This study proposes the development of a potential evaluation tool for One Health performance, in order to contribute to the scientific measurement of One Health approach and the identification of gaps where One Health capacity building is most urgently needed.

**Methods:**

We describe five steps towards a global One Health index (GOHI), including (i) framework formulation; (ii) indicator selection; (iii) database building; (iv) weight determination; and (v) GOHI scores calculation. A cell-like framework for GOHI is proposed, which comprises an external drivers index (EDI), an intrinsic drivers index (IDI) and a core drivers index (CDI). We construct the indicator scheme for GOHI based on this framework after multiple rounds of panel discussions with our expert advisory committee. A fuzzy analytical hierarchy process is adopted to determine the weights for each of the indicators.

**Results:**

The weighted indicator scheme of GOHI comprises three first-level indicators, 13 second-level indicators, and 57 third-level indicators. According to the pilot analysis based on the data from more than 200 countries/territories the GOHI scores overall are far from ideal (the highest score of 65.0 out of a maximum score of 100), and we found considerable variations among different countries/territories (31.8–65.0). The results from the pilot analysis are consistent with the results from a literature review, which suggests that a GOHI as a potential tool for the assessment of One Health performance might be feasible.

**Conclusions:**

GOHI—subject to rigorous validation—would represent the world’s first evaluation tool that constructs the conceptual framework from a holistic perspective of One Health. Future application of GOHI might promote a common understanding of a strong One Health approach and provide reference for promoting effective measures to strengthen One Health capacity building. With further adaptations under various scenarios, GOHI, along with its technical protocols and databases, will be updated regularly to address current technical limitations, and capture new knowledge.

**Graphical Abstract:**

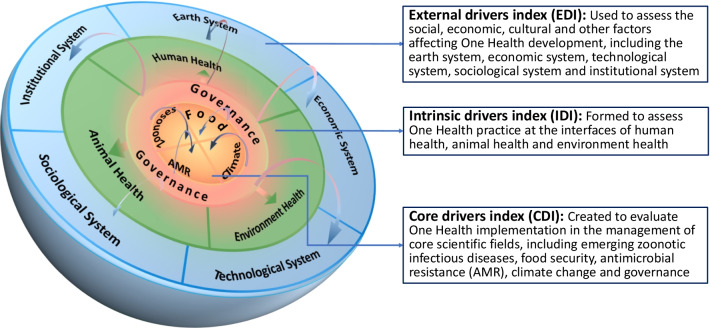

**Supplementary Information:**

The online version contains supplementary material available at 10.1186/s40249-022-00979-9.

## Background

Over the past 20 years, the world has encountered several major public health emergencies caused by zoonotic diseases, including severe acute respiratory syndrome (SARS; 2002–2004), Ebola virus disease in West Africa (2013–2016), Zika virus in the Americas (2015–2016), and the ongoing coronavirus disease 2019 (COVID-19) pandemic. Since 1970, zoonotic diseases have accounted for more than 75% of emerging and re-emerging infectious diseases, leading to 2.5 billion infections and 2.7 million deaths each year [1]. As of the end of February 2022, there have been about 440 million confirmed cases of COVID-19, including 5.98 million deaths [2]. While humans have never possessed such a sophisticated arsenal of technologies for surveillance, prevention, treatment and management of diseases as they have available today, the threats of outbreaks of emerging and remerging zoonotic infectious diseases are very real indeed. As highlighted by the COVID-19 pandemic, the lack of cross-cooperation among human, animal and environmental/ecosystem health agencies results in great difficulty in early detection, prevention and containment of epidemics [3, 4].

A One Health approach has been suggested to address complex global health problems at the human–animal–environment interface, coupled with inter- and trans-disciplinary involvement, and transnational collaboration. On December 1, 2021, the Food and Agriculture Organization of the United Nations (FAO), the World Organization for Animal Health (OIE), the World Health Organization (WHO) and the United Nations Environment Programme (UNEP)’s One Health High-Level Expert Panel (OHHLEP) formally defined One Health as “an integrated, unifying approach that aims to sustainably balance and optimize the health of people, animals and ecosystems” [5]. In this definition, we consider that “optimize” refers to a Pareto improvement [6] of the whole system at human–animal–environment interface. Specifically, without sacrificing the benefits of any one of the three (human health, animal health and environmental health), at least one aspect or the human–animal–environment system as a whole is made better [7–9]. Or, with the same effectiveness obtained, the cost of the intervention is reduced, and hence, a higher cost-effectiveness could be achieved [10]. In such a process, a closer cooperation of different sectors is crucial.

In response to the 2002–2004 outbreak of SARS and H5N1 avian influenza, which generated global attention, the World Wildlife Conservation Association officially proposed the concept of One Health and released *the Manhattan Twelve Principles* encapsulating this approach [11]. In 2005, *The Lancet* published its first reference to “One Health” in an article about the cooperation between human and animal health to strengthen health systems [12]. In 2008, FAO, OIE, WHO, UNICEF, The World Bank and the United Nations System Influenza Coordination (UNSIC) officially suggested One Health as the approach to deal with global epidemics [13]. In 2009, funded by the Rockefeller Foundation, the One Health Commission was established with the objective of disseminating the One Health approach more widely [14]. In 2020, the OHHLEP was jointly established by FAO, OIE, WHO and UNEP, to provide expert technical guidance on key scientific issues in One Health [15]. At the behest of the international community, countries including the United Kingdom and the United States of America established specific government entities or initiatives [16, 17] to lead administrative coordination, fundraising and policy-making relevant to One Health promotion.

Based on a summary of previous literature, we consider that a One Health approach implies an integrated and unifying approach on several levels: firstly, it unifies human health, animal health and environment health with the aim of achieving the Pareto improvement for human–animal–environment systems [18, 19]; secondly, it mobilizes cross-sectoral and multi-disciplinary cooperation by breaking down barriers in governance [20]; thirdly, it promotes joint cooperation between countries and regions to tackle threats at global, regional and local levels, such as pandemic preparedness and response, climate change, etc. [21]; and fourthly, it promotes broader societal participation and better societal awareness for the sustainable development of human–animal–environment systems [22].

Although the One Health approach has been well-recognized [23], its determinants and practical enablers have yet to be clarified. The dearth of real-world evidence has hindered the identification of gaps in the human–animal–environment health nexus, which hampers the application of a One Health approach in shaping policies and practice. A well-constructed conceptual system, along with an appropriate evaluation scheme, is needed to better understand the current situation and to set goals and strategies for One Health implementation, readily tailored to specific social-ecological settings.

Recognizing this challenge, we propose five steps towards development of a global One Health index (GOHI) as a potential tool for the systematic evaluation of One Health performance. In a next step, our proposed GOHI needs to be validated. A rigorously validated GOHI might then help improve the application of a One Health approach globally. We hope that our proposal and frame of a GOHI will be used to promote effective measures to fill the gaps in One Health practice of each country/territory and promote a wider application of One Health approaches in shaping real-world policies and practices.

In this paper, we describe the essentials of One Health, propose a conceptual framework for GOHI and construct a weighted indicator scheme. With the adaptation of a mixed-methods approach that combines a qualitative assessment with quantitative evaluation, we build up the GOHI database and test the GOHI tool with a pilot analysis on the global performance of One Health.

## Methods

**Fig. 1:**
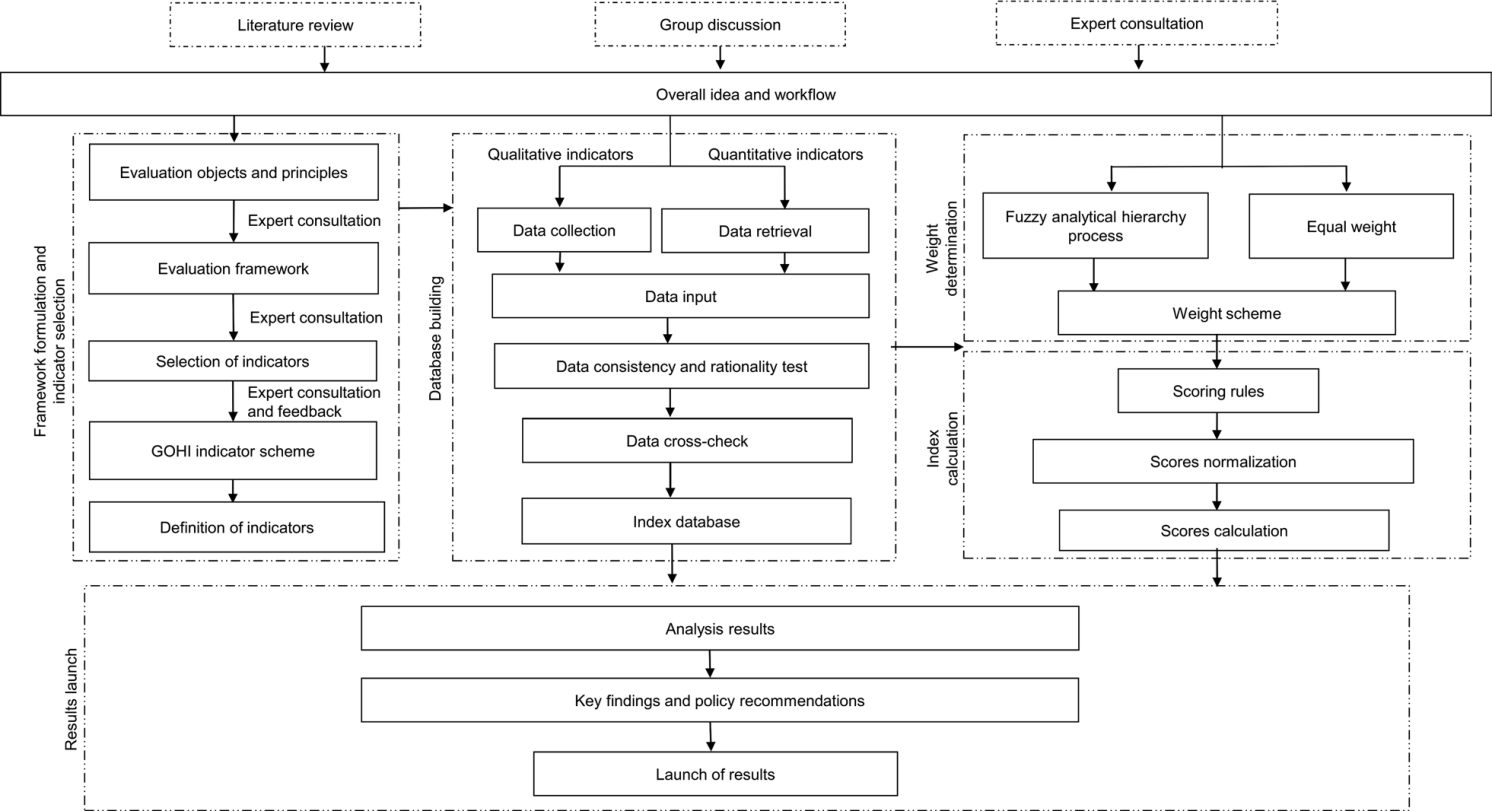
Flowchart for the construction of a global One Health index (GOHI)

GOHI is constructed in five steps, including (i) framework formulation; (ii) indicator selection; (iii) database building; (iv) weight determination; and (v) GOHI scores calculation (Fig. 1).

### Expert advisory committee

To ensure the reliability of the index, we are committed to incorporating the opinions of experts and have established a GOHI expert advisory committee (Table 1).

**Table 1:** Characteristics of the expert advisory committee assisting with the development of a global One Health index (GOHI) [n=29]

Item	Category	Counts	Percentage (%)
Gender	Male	16	55.2
	Female	13	44.8
Age (years)	21–30	6	20.7
	31–40	12	41.4
	41–50	8	27.6
	> 50	3	10.3
Education	Doctoral degree	20	69.0
	Master degree	9	31.0
	Bachelor degree	0	0.0
	College degree	0	0.0
	Other	0	0.0
Professional level	Senior level	10	34.5
	Vice-senior level	8	27.6
	Middle level	9	31.0
	Primary level	2	6.9
Type of work	Medical institutions	11	37.9
	Colleges and Universities	15	51.7
	Governments	3	10.3
Working experience (in years)	< 10 years	14	48.3
	10–20 years	8	27.6
	> 20 years	7	24.1
Primary research area	Human medicine	12	41.4
	Veterinary science	7	24.1
	Environmental science	7	24.1
	Social science	1	3.4
	Political science	1	3.4
	Management science	1	3.4
Secondary research area	Human medicine	7	24.1
	Veterinary science	1	3.4
	Environmental science	1	3.4
	Social science	3	10.3
	Political science	3	10.3
	Management science	5	17.2

*The committee expansion is under way

The School of Global Health, Chinese Center for Tropical Diseases Research, Shanghai Jiao Tong University School of Medicine, maintains a database of cooperating partners with expertise in relevant research fields. To construct the GOHI expert advisory committee, we selected 10 experts from the expert database by convenience sampling based on professional expertise, research relevance and willingness to participate. Then, we continued to include more experts through snowball sampling, and eventually built up an expert advisory committee of 29 experts from the areas of human medicine, veterinary science, environmental science, social science, political science and management science.

### Index framework formulation

In developing the index framework, we assume three essential components of a One Health approach. Firstly, One Health recognizes the close links among human health, animal health and environment health, and mobilizes multiple sectors, disciplines and communities at varying levels of society; secondly, the use of a One Health approach represents more holistic thinking. In the process of dealing with increasingly complex global health problems, many factors, such as social, cultural, economic and natural factors, should be considered, especially their influence on the human–animal–environment nexus; thirdly, a One Health approach promotes the coordinated development of human–animal–environment systems and improve the ability to more effectively address global challenges.

Based on these essentials of One Health, we use a three-layer structure of external, intrinsic and core layers as the basis of our proposed evaluation framework, which implies structure–process–outcome thinking [24]. The external layer of One Health is to provide a facilitating environment for One Health’s development; the intrinsic layer of One Health composes elements for the integrated development of human–animal–environment system; and the core layer consists of components in the response to the challenges of One Health key issues.

**Fig. 2:**
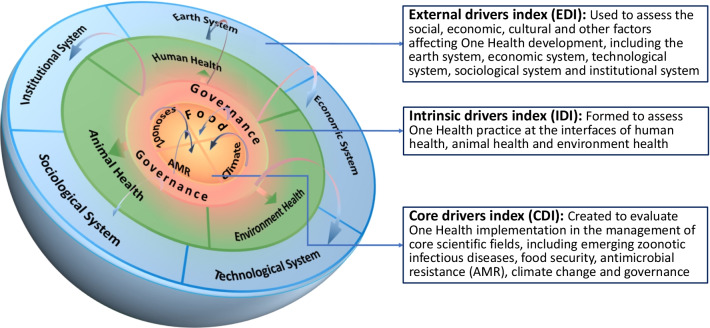
Cell-like framework of global One Health index (GOHI)

Therefore, a cell-like framework of GOHI (Fig. 2) has been developed, which comprises an external drivers index (EDI), an intrinsic drivers index (IDI) and a core drivers index (CDI). The EDI is used to assess the social, economic, cultural and other factors affecting One Health development, including the earth system, economic system, technological system, sociological system and institutional system [25]. The IDI is formed to assess One Health practice at the interfaces of human health, animal health and environment health. The CDI is created to evaluate One Health implementation in the management of core scientific fields, including emerging zoonotic infectious diseases, food security, antimicrobial resistance (AMR), climate change and governance.

The selection of the core scientific fields of CDI is mainly based on literature reviews. The report entitled “Contributing to One World, One Health: a strategic framework for reducing risks of infectious diseases at the animal-human-environment interface” jointly released by FAO, OIE, WHO, UNICEF, The World Bank and the UNSIC [26], has identified these core issues at the animal–human–ecosystem interface, including zoonoses, food security, AMR and climate change. The journal *Nature* published a comment put forth by Kahn [18], which took environmental hazards, infectious diseases and AMR as typical applications of One Health. Another comment, recently published in *The Lancet* [27], summarized key dimensions of One Health as shared environment, safe food and food systems and shared medicines and intervention, and proposed that these challenges could be further impacted by climate change. Moreover, the role of governance in establishing interdisciplinary and interdepartmental mechanisms for the integrated development of human–animal–environment system has also been elaborated in the literature [28].

### Indicator selection

The indicators are selected according to the principles of relevance, authoritative sources, open access, completeness, timeliness, comparability and country-level data (Table 2). Based on the established cell-like framework, we have conducted four rounds of expert advisory committee consultations and several key infomant interviews with experts from multiple UN agencies, including four experts from WHO, two from The World Bank and one each from FAO and World Meteorological Organization (WMO). In the first round, we determined the core data sources based on the main principles for constructing the indicator scheme, and started a large-scale collection of data for indicators. In the second round, we reorganized the indicator scheme based on the collected indicators, and determined the frame of the first-, second- and third-level indicators. In the third round, we explored strategies for integrating indicators and condense the number of indicators. In the fourth round, we discussed the demonstration of the agreed indicator scheme.

**Table 2:** Data selection criteria for developing a global One Health index (GOHI)

Principle	Criteria
Relevance	Data should represent the content of corresponding indicators
Authoritative sources	Data are retrieved from authoritative global/countries agencies
Open access	Data are available from public open sources with transparent collecting/statistical methods and high level of integrity
Completeness	Data used for indicators should cover a sufficient number of countries/territories
Timeliness	Data should cover recent temporal period and are updated annually
Comparability	For single indicator, data should be measured with an established and unified method and peer-reviewed across countries/territories
Country-level data	Data should describe the status of indicators at country-level

### Database building

Data generated between 2016 and 2020 were collected for each indicator and used to construct the GOHI database. Our data were mainly extracted from authoritative databases (Table 3). We also developed a few self-designed indicators which were constituted by structured questions, the answers to which were found from results of searching websites or open-access sources by our research team. To ensure quality of the data, we performed cross-checking and consistency testing for data collection.

**Table 3:** Data sources utilized for the development of a global One Health index (GOHI)

Category	Sources
External drivers index (EDI)	Databases from FAO, Organization for Economic Cooperation and Development (OECD), The World Bank, Our World in Data, International Energy Agency (IEA), International Telecommunication Union (ITU)
Intrinsic drivers index (IDI)	Databases from Sustainable Development Goals (SDGs), WHO-The Global Health Observatory, IHME-Global Burden of Disease (GBD), The World Bank, Environmental Performance Index (EPI), FAO, Global Ocean Health Index scores, Our World in Data
Core drivers index (CDI)	
Governance	Data from SDGs The World Bank, government website portal
Zoonotic diseases	Data from WHO, OIE, The World Bank, Global Health Security Index (GHS), GHDx
Food security	Data from FAO, The World Bank, WHO, UN, UNHCR, UNEP
Antimicrobial resistance	Data from global antimicrobial resistance and use surveillance system (GLASS), GHS, Tripartite AMR Country Self-Assessment Survey (TrACSS), European Centre for Disease Prevention and Control, The Pan American Health Organization (PAHO)/WHO
Climate change	Data from The World Bank, Lancet Countdown, Our World in Data, OECD.Stat

### Weight determination

A fuzzy analytical hierarchy process (FAHP) [29] was adopted to assign the weights for most of the indicators. We used a fuzzy comparison matrix based on the judgments of different experts to generate the weight matrix of indicators. We conducted two rounds of investigations among our expert advisory committee and collected the experts’ opinions by several rounds of interrogation on the comparison of relative importance between indicators. The details on weight determination are described in Additional file 1.

### Data preprocessing

For missing data, we interpolated missing values using the mean of the counterparts from three countries with the most similar conditions. If the data of the indicator were missing for more than 160 countries, the indicator was excluded from the calculation; if the country’s data missing rate exceeded 50% in EDI, IDI or any of the five dimensions in CDI (governance, zoonotic diseases, food security, AMR and climate change), the country was excluded from the calculation.

The choice of the missing rate threshold for the inclusion of countries/territories in GOHI was made based on the consideration of prevalent data incompleteness and the number of countries/territories that could be included. GOHI devotes to include as many countries/territories as possible as it aims to provide reference for every country/territory around the world in One Health promotion. However, due to inherent data scarcity, if strict threshold of data missing rate (e.g., 15%) was adopted, most countries/territories would be excluded (in our database, with the threshold of 50% data missing rate, the number of countries/territories included was 146; with the threshold of 15% data missing rate, the number of countries/territories included would have been reduced to only 6). Therefore, we chose a data missing rate of 50% as the inclusion threshold.

For those indicators with values of 0 or 1, we took measures to deal with the bias from over-polarization. For the value 0, we replaced it with a positive number sampled from the normal distribution ***N***(0, 0.16^2^); for the value 1, we replaced it with a number less than 1 sampled from the normal distribution ***N***(1, 0.16^2^). By using the method of continuous processing of discrete random variable, the possible bias from some indicators with only 0 or 1 can be reduced when calculating the weights of our GOHI indicators.

### GOHI score calculation

We believe that good One Health performance requires the following: it needs to have a facilitating external environment for development, which should be indicated by a high EDI score; meanwhile, it needs to perform well in the three areas of human health, animal health and environmental health, which should drive a high IDI score; moreover, it needs to be able to cope with the challenges in core issues of a One Health approach, including zoonosis prevention and control, achieving food security, managing AMR, mitigating and adapting to climate change, which–collectively–would be indicated by a high CDI score.

Hence, based on the logic of evaluating One Health performance, the EDI, IDI and CDI scores are weighted and summed to obtain the final total score of GOHI.

For each sub-indicator, we set the best/worst value for it, and used the following equation to normalize the sub-indicator [30, 31]:

$${S}_{ij}=\left\{\frac{\begin{array}{l}0\\ {X}_{ij}-{X}_{worst,j}\end{array}}{\begin{array}{c}{X}_{best,j}-{X}_{worst,j}\\ 100\end{array}}\times 100\right.$$

where ${S}_{ij}$ denotes the normalized score for j-th sub-indicator of i-th country; ${X}_{ij}$ denotes the original values for j-th sub-indicator of i-th country; ${X}_{best,j}$ denotes the original values of best performance for j-th sub-indicator; ${X}_{worst,j}$ denotes the original values of worst performance for j-th sub-indicator.

The weighted sum of the scores of the lower level indicators was derived from the following equation to obtain the scores of the upper level indicators:

$${Indicator\, score}_{ih}=\sum_{{1}_{h}}^{{m}_{h}}{S}_{{ij}_{h}}\times {W}_{{j}_{h}} , \sum_{{1}_{h}}^{{m}_{h}}{W}_{{j}_{h}}=1$$

where ${m}$ denotes the total number of the sub-indicators under h-th indicator; ${j}_{h}$ denotes the j-th sub-indicator under h-th indicator; ${S}_{{ij}_{h}}$ denotes the score of ${j}_{h}$-th sub-indicator in i-th country; ${W}_{{j}_{h}}$ denotes the weight of ${j}_{h}$-th sub-indicator.

### Framework validation

We used the most recently available data from GOHI database to conduct a pilot analysis of One Health performance at the global, regional and dimensional level. We compared the results from GOHI with findings from the literature, in order to validate the feasibility of our tool.

## Results

### GOHI indicator and weight scheme

**Fig. 3:**
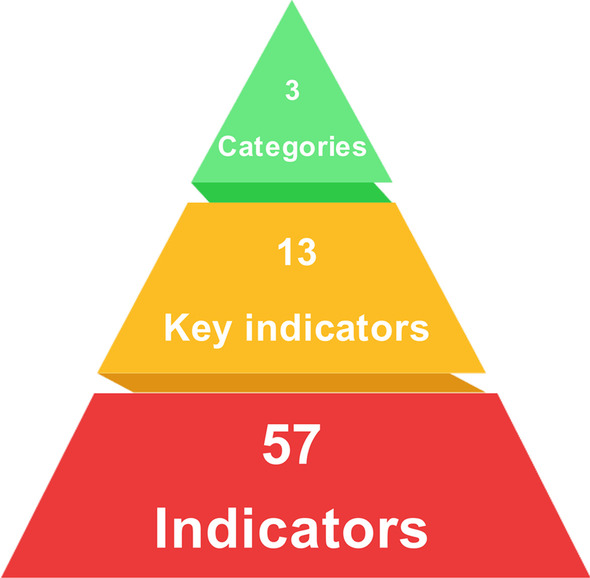
Indicator structure of a global One Health index (GOHI). Categories, Key indicators and Indicators in the figure are comparable to the first-level, second-level and third-level indicators presented in the text, respectively

The indictor scheme for GOHI comprises of three first-level indicators, 13 second-level indicators and 57 third-level indicators (Fig. 3). The first- and second-level indicators were determined based on our cell-like framework; the third-level indicators were determined from an extensive literature review and panel discussions with our expert advisory committee. For the indicators A1‒A5, B1‒B3, C3.1‒C3.5 and C4.1‒C4.5, equal weights were assigned as they are assumed to be equally important as their peer indicators; for the other indicators, FAHP was used to determine the weights assigned. Table 4 shows the indicator and weight scheme of GOHI. The full details of the indicators and weights of GOHI are shown in Additional file 2.

**Table 4:** Indicator and weight scheme of our proposed global One Health index (GOHI)

Category	Weight (%)	Key indicator	Weight (%)	Indicator	Weight (%)
A External drivers index (EDI)	15.2	A1 Earth system	20.0	A1.1 Land	18.7
				A1.2 Forest	18.3
				A1.3 Water	23.7
				A1.4 Air	22.8
				A1.5 Natural disasters	16.6
		A2 Institutional system	20.0	A2.1 Justice	45.5
				A2.2 Governance	54.5
		A3 Economic system	20.0	A3.1 Finance	37.7
				A3.2 Work	30.4
				A3.3 Housing	31.9
		A4 Sociological system	20.0	A4.1 Demography	33.0
				A4.2 Education	37.7
				A4.3 Inequalities	29.3
		A5 Technological system	20.0	A5.1 Transport	30.8
				A5.2 Technology adoption	35.1
				A5.3 Consumption and production	34.1
B Intrinsic drivers index (IDI)	16.3	B1 Human health	33.3	B1.1 Reproductive, maternal, new-born and child health	20.6
				B1.2 Infectious diseases	19.5
				B1.3 Non-communicable diseases and mental health	15.9
				B1.4 Injuries and violence	13.5
				B1.5 Universal health coverage and health systems	17.5
				B1.6 Health risk	13.0
		B2 Animal health and ecosystem diversity	33.3	B2.1 Animal epidemic diseases	31.9
				B2.2 Animal welfare	24.7
				B2.3 Animal nutritional status	17.4
				B2.4 Animal biodiversity	26.1
		B3 Environmental health	33.3	B3.1 Air quality and climate change	23.8
				B3.2 Land resources	19.6
				B3.3 Sanitation and water resources	20.7
				B3.4 Hazardous chemicals	17.5
				B3.5 Environmental biodiversity	18.4
C Core drivers index (CDI)	68.5	C1 Governance	21.7	C1.1 Participation	11.0
				C1.2 Rule of law	15.8
				C1.3 Transparency	10.0
				C1.4 Responsiveness	12.6
				C1.5 Consensus oriented	10.8
				C1.6 Equity and inclusiveness	13.8
				C1.7 Effectiveness and efficiency	13.2
				C1.8 Political support	12.9
		C2 Zoonotic diseases	20.3	C2.1 Source of infection	23.7
				C2.2 Route of transmission	25.3
				C2.3 Targeted population	19.1
				C2.4 Capacity building	16.8
				C2.5 Outcomes (case-studies)	15.1
		C3 Food security	21.4	C3.1 Food demand and supply	20.0
				C3.2 Food safety	20.0
				C3.3 Nutrition	20.0
				C3.4 Natural and social circumstances	20.0
				C3.5 Government support and response	20.0
		C4 Antimicrobial resistance	18.1	C4.1 AMR surveillance system	20.0
				C4.2 AMR laboratory network and coordination capacity	20.0
				C4.3 Antimicrobial control and optimization	20.0
				C4.4 Improve awareness and understanding	20.0
				C4.5 AMR rate for important antibiotics	20.0
		C5 Climate change	18.5	C5.1 Government response	37.9
				C5.2 Climate change risks	29.6
				C5.3 Health outcome	32.5

### GOHI pilot analysis

**Fig. 4:**
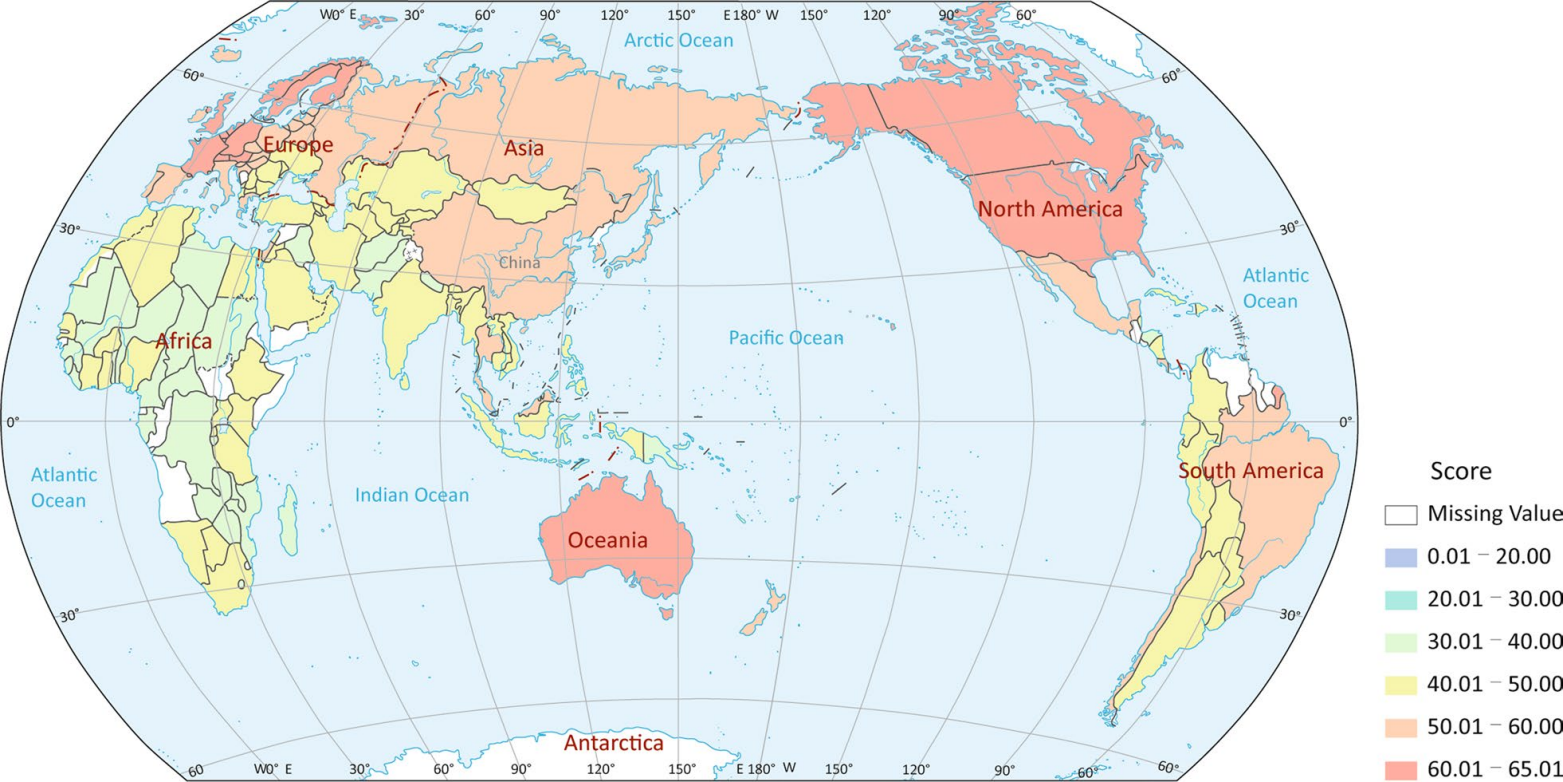
Global score map of a global One Health index (GOHI). Map approval No. GS (2022) 1516. The different colors represent different GOHI score ranges, as shown in the legend

We calculated the GOHI score for the countries/territories included, and show different ranges in performance in different colours on the map in Fig. 4. Overall, the GOHI scores of countries/territories worldwide are far from optimal (100 points), with the highest score of 65.0 (Sweden). The highest score range of 60 and above mainly includes countries in North America, Europe and Oceania, while countries in Africa obtained considerably lower scores in the range of 30‒50.

**Fig. 5:**
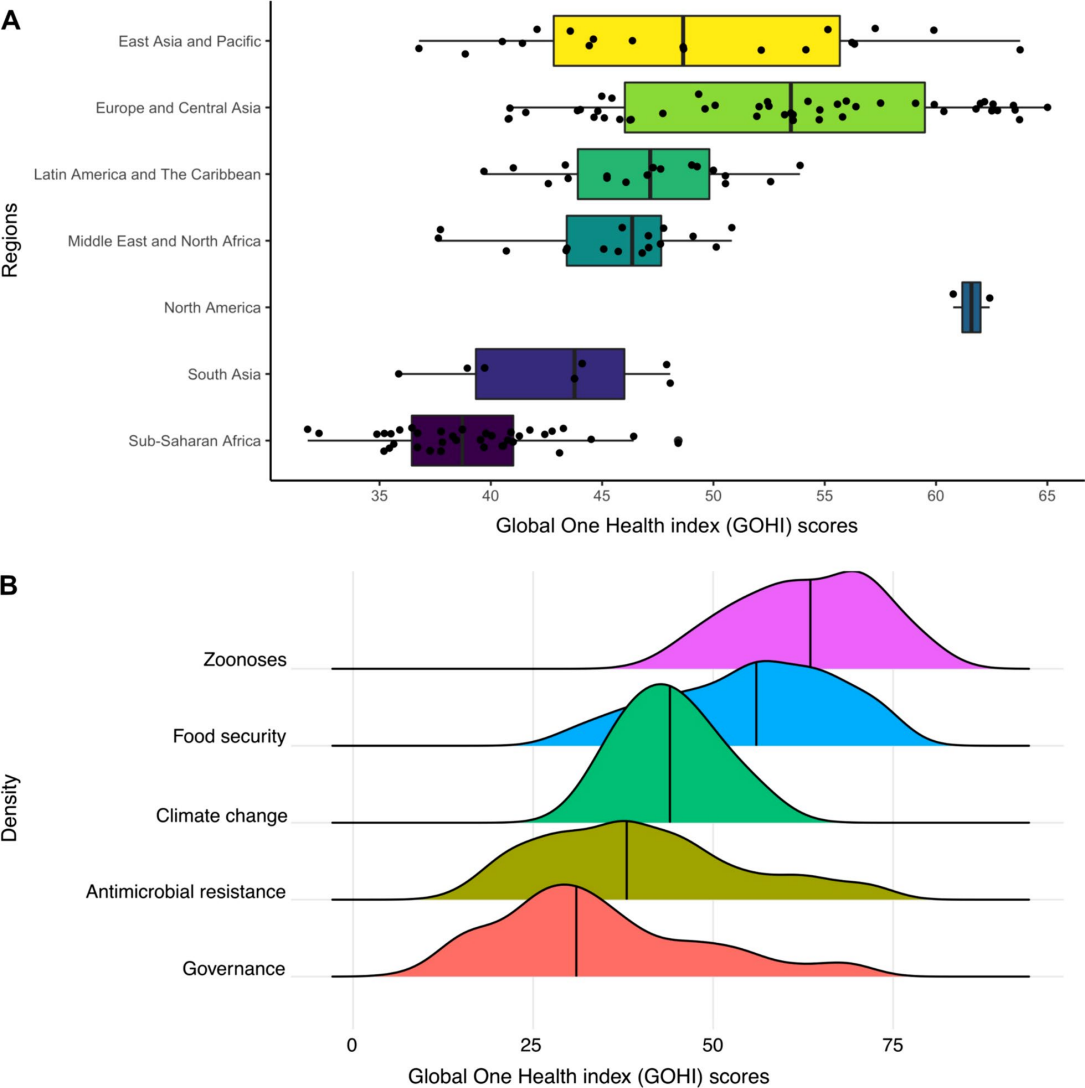
Regional (**A**) and dimensional (**B**) score distribution of a global One Health index (GOHI). **A** Boxplots of GOHI scores across countries by regions. **B** Density plots of subgroup GOHI scores across countries by five dimensions of core drivers index (CDI)

Figure 5A shows that the GOHI scores vary considerably by regions. The regions with GOHI scores, ranked according to their median (median; lower–upper bound), are as follows: North America (61.6; 60.8–62.4), Europe and Central Asia (53.5; 40.8–65.0), East Asia and Pacific (48.6; 36.8–63.8), Latin America and the Caribbean (47.2; 39.7–53.9), Middle East and North Africa (46.4; 37.6–50.8), South Asia (43.8; 35.9–48.1) and sub-Saharan Africa (38.7; 31.8–48.4). North America has the highest median, while sub-Saharan African has the lowest.

Within our framework, the CDI has five dimensions. Figure 5B shows that the dimensions of CDI with the GOHI scores ranked, according to their median, were as follows: zoonotic diseases (63.7), food security (56.3), climate change (43.6), AMR (37.8) and One Health governance (31.5). In addition, for AMR and One Health governance, the distributions of scores were wider, indicating that a large variation exists in the scores among countries/territories.

**Fig. 6:**
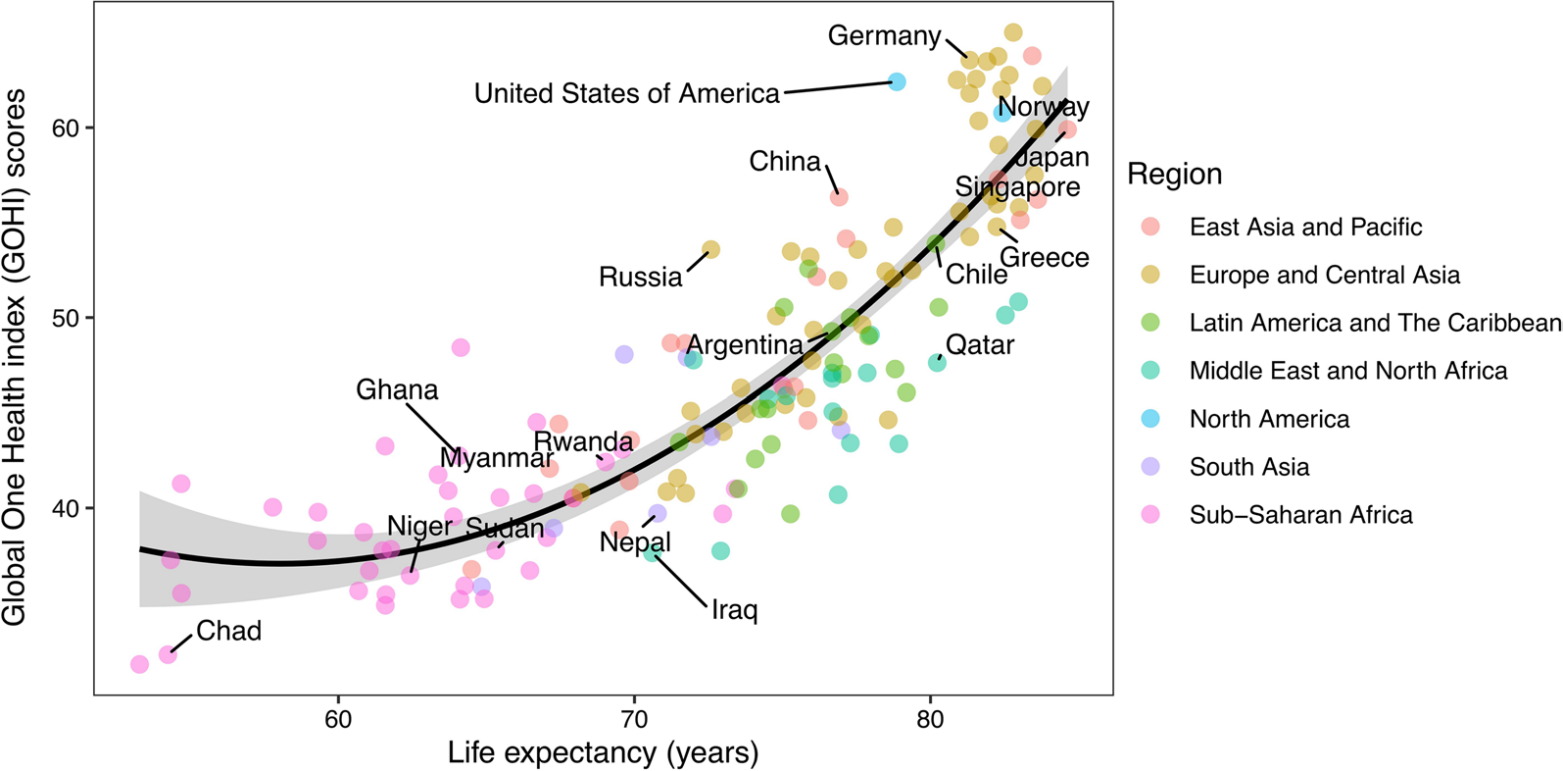
Correlation analysis of life expectancy and a global One Health index (GOHI) score. The relationship between national GOHI scores and average life expectancy fits the quadratic regression model with adjusted R^2^ = 0.76 (*P* < 0.001)

We also conducted a correlation analysis on GOHI score and average life expectancy of each country/territory. Figure 6 shows that the relationship between country GOHI score and average life expectancy was fitted well by a quadratic regression model (*r* = 0.76, *P* < 0.001).

## Discussion

### Towards a GOHI

The issues highlighted by our pilot analysis, including suboptimal global development, significant regional variations and weak governance capabilities in One Health, are consistent with the literature. This suggests that GOHI is capable of reflecting the current status of One Health development.

The FAO, OIE, UNEP and WHO are working together to mainstream One Health in order to better respond to global health threats and promote sustainable development. There is wide variation in other applications of One Health practices at national level. In 2014, the European Cooperation in Science and Technology funded the Network for Evaluation of One Health, dedicated to providing quantitative evaluation tools and evidence for One Health-related research and practices [16]. In 2019, the United States of America set up the One Health steering committee under FDA, officially responsible for coordinating and organizing One Health research and practice [32]. From 2021, the German government allocated 150 million Euro per annum to facilitate cross-sectoral and international cooperation in One Health [33]. Led by the Shanghai Jiao Tong University-The University of Edinburgh One Health Center and other institutions [34], China is also actively participating in the promotion of the One Health concept in health governance. In many regions, such as sub-Saharan Africa, the One Health approach has yet to be applied by policymakers.

The world is experiencing a series and overlapping challenges arising from zoonotic diseases, climate change, AMR, food insecurity, to name but a few. There is a pressing need for all countries to promote a One Health approach in decision-making practices. Weaknesses in managing AMR and in One Health governance illustrated in the pilot analysis underscore concerns identified by scholars in previous studies. According to the Tripartite AMR Country Self-Assessment Survey, jointly established by FAO, WHO and OIE in 2021 [35], 153 countries around the world have issued national action guidelines on AMR, but only 95 countries have implemented practices according to the guidelines, and more than 70 countries still lack formal cross-sectoral governance or coordination mechanisms on AMR.

The correlation analysis between national GOHI and average life expectancy highlights the potential for application of a GOHI. As an assessment tool, GOHI may be applied to predict the effect of interventions and health outcomes, which will benefit the prioritization of resource input and formulation of relevant strategies.

### Potential of a GOHI

With growing needs for effective decision making, there have been several global studies on quantitative evaluation of One Health. For example, the Nuclear Threat Initiative (NTI) and Center for Health Security of the Johns Hopkins Bloomberg School of Public Health jointly developed the global health security index, which assessed 195 countries/territories for several aspects, including prevention, detection and reporting, rapid response, health system, compliance with international norms and risk environment [36]; Yale Center for Environmental Law and Policy (YCELP) and Center for International Earth Science Information Network Earth Institute jointly developed an environmental performance index, and ranked 180 countries on 32 performance indicators of environmental health and ecosystem vitality [31]. However, these studies have primarily focused on one of the components of One Health, rather than the bundle that constitutes One Health.

A number of published reports have discussed the conceptual framework for One Health evaluation. Rüegg et al. [37] analyzed the logical relationship among the elements of One Health, using a system dynamics approach and proposed a formula for the quantification of One Health. Wang et al. [38] established criteria for prioritizing zoonotic diseases, which included disease hazard/severity, epidemic scale and intensity, economic impact, prevention and control, and social impact. However, these studies have focused on the exploration of the One Health evaluation framework and methodology, without using global-level empirical data for analysis.

GOHI is the world’s first research tool that builds an evaluation framework from a holistic perspective of One Health and uses data from more than 200 countries and territories for empirical analysis. Unlike the existing global databases, which either use structured questionnaire scores (e.g. global health security index) or exclusively quantitative data (e.g. environmental performance index), GOHI combines qualitative and quantitative data with a mixed-methods approach in data collection and analysis, which makes GOHI more flexible and applicable to different situations in different countries.

### Potential function of GOHI

Firstly, the introduction of the cell-like framework of GOHI provides a clear definition and promotes common understanding of the determinants and functioning of a strong One Health approach, which will serve as the conceptual foundation for policy making. Moreover, the GOHI database enhances the consistency and quality of One Health systems, including via collaboration in data transparency and information sharing.

Secondly, GOHI is able to serve as a tool for the assessment of the performance of One Health approaches at global, continental, country and sub-regional levels. It provides a means for identifying gaps and promoting the adoption of effective measures to strengthen One Health capacity building in countries/territories where it is needed most. GOHI sets out to distill examples of best practice and guides countries to identify potential weaknesses in One Health implementation. This in turn helps identify priorities for international assistance in One Health issues, which is much needed for poverty alleviation and achievement of the SDGs.

Thirdly, the CDI in GOHI focuses on emerging zoonotic infectious diseases, AMR, food security and climate change. All of the countermeasures tackling these global challenges need further development and new technologies, such as surveillance and response technologies, preparedness and early warning systems, prediction and modelling platforms. GOHI aims to support early identification of the gaps in technology development in relevant fields, particularly in the fields of infectious disease control and prevention, emergency response and preparedness and AMR monitoring and control, which are core issues in One Health practice.

### Limitations

There are several limitations of our study. Firstly, in order to ensure the validity of data, we use publicly available global official data as the main data sources for GOHI, which may have resulted in limitations on the inclusion of some indicators. Due to lack of data, some sensitive indicators (such as animal disease incidence, animal disease burden, animal vaccine usage, etc.) have not been included in the analysis. In addition, when searching for data from self-designed indicators in different countries, we use English and French as the main search languages, which may introduce collection bias.

Secondly, the experts in our advisory expert committee listed in Table 1 are geographically from China, which may limit the global representativeness of the committee. Nevertheless, our research team includes several experts from international organizations and research institutions outside of China and we have conducted several key informant interviews with experts from various UN agencies during the research process, which have brought about some international perspective to our research. In the next stage of GOHI, we will include more international experts to participate in our research, in order to improve the global representativeness of our expert committee.

Thirdly, our GOHI validation is a preliminary step, which we use as a case study to illustrate potential applications. Our proposed GOHI now needs rigorous validation. For example, we may form a consensus parameter based on data modelling for the judgement of consistency between GOHI scores and previous published work on One Health.

Moreover, in follow-up research, based on the framework and database of GOHI, we plan to conduct further investigations on data mining and extensions of mathematical models, in order to analyze the performance of One Health approach in typical real-world scenarios (e.g. zoonoses control) and to what extent Pareto improvement is achieved in pursuing the overall benefit of human–animal–environment systems.

## Conclusions

With the growing recognition of the intimate links of human health, animal health and environment health, a One Health approach is being increasingly applied in decision-making. The formulation of a consolidated evaluation tool is essential for the valid assessment of One Health approaches. Through consulting an expert advisory committee and the adaptation of a mixed-methods approach, we have developed a cell-like framework and a weighted three-level indicator scheme for GOHI, which needs broader validation beyond the GOHI database pursued here.

It is anticipated that GOHI along with its technical protocols, will not remain static but will evolve with more applications in research, practice and policy. Through the establishment of this tool, we aim to contribute a foundation for further improvement and rigorous validation. Moreover, the construction of GOHI has highlighted the significance of producing higher quality global One Health database, and of sharing the data with interested constituencies.

While the application of One Health approaches relies on strong political commitment, GOHI will support the implementation of One Health at national, regional and global levels. Thus far, very few countries have established a government agency specifically responsible for coordination of a One Health approach. The lack of governance mechanisms is still the biggest bottleneck in capacity building for One Health at varying levels. There is a need for a more conducive political environment to promote One Health applications, and to stimulate multi-sectoral and trans-disciplinary cooperation at global, regional and national scales.

## Supplementary Information

**Additional file 1.** Weights calculation of global One Health index (GOHI).

**Additional file 2. **The indicators and weights for establishment of global One Health index (GOHI).

## Data Availability

The full study protocol and the datasets are available, following manuscript publication, upon request from the corresponding author (Xiao-Nong Zhou, zhouxn1@chinacdc.cn).

## Abbreviations

GOHI: Global One Health index
EDI: External drivers index
IDI: Intrinsic drivers index
CDI: Core drivers index
FAHP: Fuzzy analytical hierarchy process
SARS: Severe acute respiratory syndrome
COVID-19: Coronavirus disease 2019
FAO: Food and Agriculture Organization of the United Nations
OIE: World Organization for Animal Health
WHO: World Health Organization
UNEP: United Nations Environment Programme
OHHLEP: One Health High-Level Expert Panel
UNSIC: United Nations System Influenza Coordination
AMR: Antimicrobial resistance
OECD: Organization for Economic Cooperation and Development
IEA: International Energy Agency
ITU: International Telecommunication Union
SDGs: Sustainable Development Goals
IHME: Institute for Health Metrics and Evaluation
GBD: Global burden of disease
EPI: Environmental performance index
GHS: Global health security index
GLASS: Global antimicrobial resistance and use surveillance system
TrACSS: Tripartite AMR country self-assessment survey
FDA: Food and Drug Administration
NTI: Nuclear threat initiative
YCELP: Yale Center for Environmental Law and Policy
