# Supplementary material for: Towards a global One Health index: a potential assessment tool for One Health performance
Source: Infect Dis Poverty. 2022 May 22;11:57. doi: 10.1186/s40249-022-00979-9 (PMC9124287; doi:10.1186/s40249-022-00979-9)
Supplement: Supplementary file 1 — Additional file 1. Weights calculation of global One Health index (GOHI). [file 40249_2022_979_MOESM1_ESM.docx]

**Additional file 1：**

Weights calculation of global One Health index (GOHI)

1. Weights Calculation of the GOHI Indicators
2. First, according to the set of indicators, the importance judgment questionnaire between the indicators is generated by comparing each two indicators. Then experts fill in the questionnaire to select the relatively important one of the two indicators. If there are two indicators, only one comparison is required. If there are *m* indicators, the number of comparisons is (*m**(*m*-1)/2 times. The example of the importance judgment questionnaire between the first-level indicators is shown in Figure 1.

| 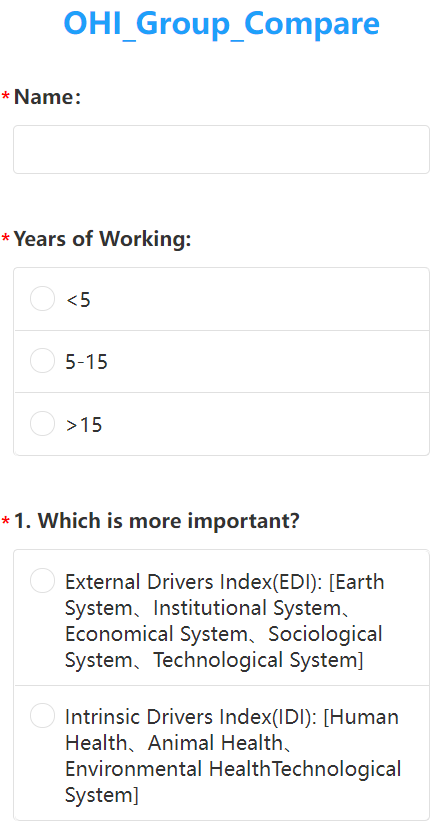 |
| --- |
| Figure 1：The example of the importance judgment questionnaire between the first-level indicators |

1. Send the indicator importance judgment questionnaire generated in the first step to as many experts as possible. According to the statistics of the judgment results, the judgment results of each expert can get a judgment matrix $R_{m\times m}^{n}$ as the formula (1). *m* indicates that there are *m* indicators. *n* means that there are *n* experts. Therefore, *n* experts obtain *n* judgment matrices ***R*** correspondingly.

| $\boldsymbol{R}_{m\times m}^{n}=\left[ \begin{matrix} r_{11} & r_{12} & \cdots& r_{1m} \\ r_{21} & r_{22} & \cdots& r_{2m} \\ \vdots& \vdots& \ddots& \vdots\\ r_{m1} & r_{m2} & \cdots& r_{mm} \end{matrix} \right]=(r_{ij}),$  $i=1,2,3\cdots,m; j=1,2,3\cdots,m; n\in\mathbf{N}^{\boldsymbol{+}}.$ | （1） |
| --- | --- |

In formula (1), $r_{ij}$ represents the judgment result of the importance of indicator *i* relative to indicator *j*, and the judgment matrix ***R*** satisfies the following properties:

1) If *i*≠*j* and the indicator *i* is more important than indicator *j*, then *r_ij_*＝1, *r_ji_*＝0;

2) If *i*＝*j*, then *r_ij_*＝*r_ji_*＝0.5.

1. Combine the judgment matrices of *n* experts obtained in the second step into a judgment matrix $\boldsymbol{R}^{'}$.

| $\boldsymbol{R}^{\boldsymbol{'}}=\frac{\left( \boldsymbol{R}^{1}+\boldsymbol{R}^{2}+\cdots+\boldsymbol{R}^{n} \right)}{n}=\left[ \begin{matrix} r_{11}^{'} & r_{12}^{'} & \cdots& r_{1m}^{'} \\ r_{21}^{'} & r_{22}^{'} & \cdots& r_{2m}^{'} \\ \vdots& \vdots& \ddots& \vdots\\ r_{m1}^{'} & r_{m2}^{'} & \cdots& r_{mm}^{'} \end{matrix} \right](r_{ij}^{'}),$  $i=1,2,3\cdots,m; j=1,2,3\cdots,m; n\in\mathbf{N}^{\boldsymbol{+}}.$ | （2） |
| --- | --- |

1. Through formula (3) and formula (4), convert the judgment matrix $\boldsymbol{R}^{'}$ obtained in the third step into a fuzzy judgment matrix ***F***.

| $r_{i}=\sum_{k=1}^{m} \left( r_{ik} \right)$ | （3） |
| --- | --- |
| $f_{ij}=\frac{r_{i}-r_{j}}{2m}+0.5$ | （4） |
| $\boldsymbol{F}=\left[ \begin{matrix} f_{11} & f_{12} & \cdots& f_{1m} \\ f_{21} & f_{22} & \cdots& f_{2m} \\ \vdots& \vdots& \ddots& \vdots\\ f_{m1} & f_{m2} & \cdots& f_{mm} \end{matrix} \right]=(f_{ij}), i=1,2,3\cdots,m; j=1,2,3\cdots,m.$ | （5） |

1. Through formula (6) and formula (7), use the fuzzy judgment matrix ***F*** obtained in the fourth step to calculate the normalized weights value of indicators.

| $S_{i}=\left( \prod_{k=1}^{m} f_{ik} \right)^{\frac{1}{m}}$ | （6） |
| --- | --- |
| $\overline{S_{i}}=\frac{S_{i}}{\sum_{i=1}^{m} \left( S_{i} \right)}$ | （7） |

The final normalized weights value of indicators is:

| $\boldsymbol{\omega}=\left( \overline{S_{1}}, \overline{S_{2}},\cdots,\overline{S_{m}} \right)$ | （8） |
| --- | --- |

1. Algorithm Implementation Process

| 表1：The Algorithm of the weights calculation of GOHI indicators based on FAHP | |
| --- | --- |
| Inputs：the statistics of the judgment results of each expert, the number of indicators: *m*, the number of experts: *n*. | |
| Process： | |
| 1： | Initial the judgement matrix ***R*.** |
| 2： | **for** *k* =1, 2, …, *n* **do** # input the statistics of the judgment results of each expert |
| 3： | **for** *i* =1, 2, …, *m* **do** |
| 4： | **for** *j* =1, 2, …, *m* **do** |
| 5： | **if** *i*==*j* **then** |
| 6： | $r_{ij}= r_{ij}+0.5$ |
| 7： | **else if** the indicator *i* is more important than indicator *j* **then** |
| 8： | $r_{ij}= r_{ij}+1$ |
| 9： | **else** $r_{ji}= r_{ji}+1$ |
| 10： | **end if** |
| 11： | **end for** |
| 12： | **end for** |
| 13： | **end for** |
| 14： | **for** *i* =1, 2, …, *m* **do** # obtain the judgement matrix $\boldsymbol{R}^{'}$ |
| 15： | **for** *j* =1, 2, …, *m* **do** |
| 16： | $r_{ij}= r_{ij}/n$ |
| 17： | **end for** |
| 18： | **end for** |
| 19： | Initial the row sum list $r=\left[ 0 \right]_{1\times m}$ of judgement matrix $\boldsymbol{R}^{'}$**.** |
| 20： | **for** *i* =1, 2, …, *m* **do** # obtain the fuzzy judgment matrix ***F*** |
| 21： | $r[i]=0$ |
| 22： | **for** *j* =1, 2, …, *m* **do** |
| 23： | $r[i]=r[i]+r_{ij}$ |
| 24： | **for** *i* =1, 2, …, *m* **do** |
| 25： | **for** *j* =1, 2, …, *m* **do** |
| 26： | $f_{ij}=\left( r[i]-r[j] \right)/\left( 2m \right)+0.5$ |
| 27： | **end for** |
| 28： | **end for** |
| 29： | Initial the weights matrix $\boldsymbol{\omega}=\left[ 0 \right]_{1\times m}$**.** |
| 30： | $S=\left[ 0 \right]_{1\times m}$ |
| 31： | **for** *i* =1, 2, …, *m* **do** |
| 32： | **for** *j* =1, 2, …, *m* **do** |
| 33： | $S[i]=S[i]+f_{ij}$ |
| 34： | **end for** |
| 35： | $\boldsymbol{\omega}[i]={S[i]}^{1/m}$ |
| 36： | **end for** |
| Output：normalized weights value $\boldsymbol{\omega}=\left( {S[1]}^{1/m}, {S[2]}^{1/m},\cdots,{S[m]}^{1/m} \right)$ | |

1. Reference
2. Ji-jun ZHANG. Fuzzy Analytical Hierarchy Process (FAHP)[J]. Fuzzy Systems and Mathematics, 2000, 14(2):80-88. (张吉军. 模糊层次分析法(FAHP)[J]. 模糊系统与数学,2000,14(2):80-88. DOI:10.3969/j.issn.1001-7402.2000.02.016.)
