# Supplementary material for: Towards a global One Health index: a potential assessment tool for One Health performance
Source: Infect Dis Poverty. 2022 May 22;11:57. doi: 10.1186/s40249-022-00979-9 (PMC9124287; doi:10.1186/s40249-022-00979-9)
Supplement: Supplementary file 2 — Additional file 2. The indicators and weights for establishment of global One Health index (GOHI). [file 40249_2022_979_MOESM2_ESM.docx]

**The indicators and weights for establishment of global One Health index (GOHI)**

| **Category** | **Weight（%）** | **Key Indicator** | **Weight（%）** | **Indicator** | **Weight（%）** | **Sub-indicator** | **Weight（%）** |
| --- | --- | --- | --- | --- | --- | --- | --- |
| External drivers index (EDI) | 15.23 | Earth system | 20.00 | Land | 18.69 | Country area | 25.00 |
|  |  |  |  |  |  | Cultivated area | 25.00 |
|  |  |  |  |  |  | Arable land area | 25.00 |
|  |  |  |  |  |  | Terrain ruggedness index | 25.00 |
|  |  |  |  | Forest | 18.29 | Forest area | 25.00 |
|  |  |  |  |  |  | Forest transition phase | 25.00 |
|  |  |  |  |  |  | Trees cover | 25.00 |
|  |  |  |  |  |  | Permanent deforestation | 25.00 |
|  |  |  |  | Water | 23.68 | Renewable water resources | 33.00 |
|  |  |  |  |  |  | Water dependency ratio | 33.00 |
|  |  |  |  |  |  | Water stress | 33.00 |
|  |  |  |  | Air | 22.76 | Co₂ emissions | 50.00 |
|  |  |  |  |  |  | Air pollution index | 50.00 |
|  |  |  |  | Natural disasters | 16.58 | Disasters death rate | 33.00 |
|  |  |  |  |  |  | Disaster economic loss | 33.00 |
|  |  |  |  |  |  | Disasters affected population | 33.00 |
|  |  | Institutional system | 20.00 | Justice | 45.53 | Unsentenced detainees | 20.00 |
|  |  |  |  |  |  | Property rights | 20.00 |
|  |  |  |  |  |  | Corruption perception index | 20.00 |
|  |  |  |  |  |  | Press freedom index | 20.00 |
|  |  |  |  |  |  | Affordability of justice | 20.00 |
|  |  |  |  | Governance | 54.47 | Voice and accountability | 10.00 |
|  |  |  |  |  |  | Government spending | 10.00 |
|  |  |  |  |  |  | Public social expenditure | 10.00 |
|  |  |  |  |  |  | Public education expenditure | 10.00 |
|  |  |  |  |  |  | Public health expenditure | 10.00 |
|  |  |  |  |  |  | Political stability | 10.00 |
|  |  |  |  |  |  | Government effectiveness | 10.00 |
|  |  |  |  |  |  | Regulatory quality | 10.00 |
|  |  |  |  |  |  | Rule of law | 10.00 |
|  |  |  |  |  |  | Control of corruption | 10.00 |
|  |  | Economic system | 20.00 | Finance | 37.67 | Gross domestic product | 20.00 |
|  |  |  |  |  |  | GDP deflator | 20.00 |
|  |  |  |  |  |  | Revenue excluding grants | 20.00 |
|  |  |  |  |  |  | Grants and other revenue | 20.00 |
|  |  |  |  |  |  | Adjusted GDP growth | 20.00 |
|  |  |  |  | Work | 30.44 | Labor force participation | 25.00 |
|  |  |  |  |  |  | Unemployment | 25.00 |
|  |  |  |  |  |  | Annual working hours | 25.00 |
|  |  |  |  |  |  | Youth condition | 25.00 |
|  |  |  |  | Housing | 31.89 | Own outright | 50.00 |
|  |  |  |  |  |  | Rent at reduced/subsidized price | 50.00 |
|  |  | Sociological system | 20.00 | Demography | 32.97 | Natural population growth | 20.00 |
|  |  |  |  |  |  | Life expectancy | 20.00 |
|  |  |  |  |  |  | Child and infant mortality | 20.00 |
|  |  |  |  |  |  | Total fertility rate | 20.00 |
|  |  |  |  |  |  | Urbanization | 20.00 |
|  |  |  |  | Education | 37.68 | Education enrollment | 12.50 |
|  |  |  |  |  |  | Literacy | 12.50 |
|  |  |  |  |  |  | PISA score | 12.50 |
|  |  |  |  |  |  | Science performance | 12.50 |
|  |  |  |  |  |  | Higher education | 12.50 |
|  |  |  |  |  |  | Expenditure on research | 12.50 |
|  |  |  |  |  |  | Female graduates | 12.50 |
|  |  |  |  |  |  | Researchers’ population | 12.50 |
|  |  |  |  | Inequalities | 29.35 | Gini coefficient | 20.00 |
|  |  |  |  |  |  | Palma ratio | 20.00 |
|  |  |  |  |  |  | Human development index | 20.00 |
|  |  |  |  |  |  | Poverty rate | 20.00 |
|  |  |  |  |  |  | Gender inequality index | 20.00 |
|  |  | Technological system | 20.00 | Transport | 30.82 | Railway travel | 50.00 |
|  |  |  |  |  |  | Air travel | 50.00 |
|  |  |  |  | Technology adoption | 35.13 | Internet population | 16.60 |
|  |  |  |  |  |  | Motor vehicle ownership | 16.60 |
|  |  |  |  |  |  | Mobile cellular subscriptions | 16.60 |
|  |  |  |  |  |  | Logistics performance index | 16.60 |
|  |  |  |  |  |  | Access to electricity | 16.60 |
|  |  |  |  |  |  | Share of renewable energy | 16.60 |
|  |  |  |  | Consumption and production | 34.05 | Energy consumption | 14.20 |
|  |  |  |  |  |  | Electricity consumption | 14.20 |
|  |  |  |  |  |  | Solid waste | 14.20 |
|  |  |  |  |  |  | Electronic waste | 14.20 |
|  |  |  |  |  |  | So₂ emissions | 14.20 |
|  |  |  |  |  |  | Nitrogen emissions | 14.20 |
|  |  |  |  |  |  | Non-recycled waste | 14.20 |
| Intrinsic drivers index (IDI) | 16.26 | Human health | 33.33 | Reproductive, maternal, new-born and child health | 20.63 | Maternal health | 25.00 |
|  |  |  |  |  |  | Neonatal health | 25.00 |
|  |  |  |  |  |  | Child health | 25.00 |
|  |  |  |  |  |  | Adolescent fertility | 25.00 |
|  |  |  |  | Infectious diseases | 19.53 | Tuberculosis | 20.00 |
|  |  |  |  |  |  | HIV | 20.00 |
|  |  |  |  |  |  | Malaria | 20.00 |
|  |  |  |  |  |  | Neglected tropical diseases | 20.00 |
|  |  |  |  |  |  | COVID-19 | 20.00 |
|  |  |  |  | Non-communicable diseases and mental health | 15.88 | Cardiovascular disease | 20.00 |
|  |  |  |  |  |  | Neoplasms | 20.00 |
|  |  |  |  |  |  | Diabetes mellitus | 20.00 |
|  |  |  |  |  |  | Chronic respiratory disease | 20.00 |
|  |  |  |  |  |  | Suicide | 20.00 |
|  |  |  |  | Injuries and violence | 13.49 | Road traffic | 33.33 |
|  |  |  |  |  |  | Unintentional poisoning | 33.33 |
|  |  |  |  |  |  | Homicide | 33.33 |
|  |  |  |  | Universal health coverage and health systems | 17.47 | Health coverage | 25.00 |
|  |  |  |  |  |  | Research and development Expenditure on health issues | 25.00 |
|  |  |  |  |  |  | Domestic health expenditure | 25.00 |
|  |  |  |  |  |  | Infants’ vaccination | 25.00 |
|  |  |  |  | Health risk | 13.01 | Unsafe or unimproved water, sanitation and hygiene | 33.33 |
|  |  |  |  |  |  | Household air pollution | 33.33 |
|  |  |  |  |  |  | Occupational risks | 33.33 |
|  |  | Animal health and ecosystem diversity | 33.33 | Animal epidemic disease | 31.87 | Diseases of domestic animal | 50.00 |
|  |  |  |  |  |  | Diseases of wild animal | 50.00 |
|  |  |  |  | Animal welfare | 24.66 | Overexploited or collapsed stocks Fish | 33.33 |
|  |  |  |  |  |  | Trawling or dredging fish | 33.33 |
|  |  |  |  |  |  | Discarded fish | 33.33 |
|  |  |  |  | Animal nutritional status | 17.36 | Chicken meat production efficiency | 25.00 |
|  |  |  |  |  |  | Pig meat production efficiency | 25.00 |
|  |  |  |  |  |  | Cattle production efficiency | 25.00 |
|  |  |  |  |  |  | Cattle milk production efficiency | 25.00 |
|  |  |  |  | Animal biodiversity | 26.11 | Endemic mammal species | 16.67 |
|  |  |  |  |  |  | Endemic bird species | 16.67 |
|  |  |  |  |  |  | Endemic amphibian species | 16.67 |
|  |  |  |  |  |  | Endemic reef-forming coral species | 16.67 |
|  |  |  |  |  |  | Endemic freshwater crab species | 16.67 |
|  |  |  |  |  |  | Endemic shark and ray species | 16.67 |
|  |  | Environmental health | 33.33 | Air quality and climate change | 23.82% | Ambient particulate matter pollution | 20.00 |
|  |  |  |  |  |  | Household solid fuels | 20.00 |
|  |  |  |  |  |  | Ambient ozone pollution | 20.00 |
|  |  |  |  |  |  | Climate risk | 20.00 |
|  |  |  |  |  |  | Greenhouse gas | 20.00 |
|  |  |  |  | Land resources | 19.55 | Area at risk elevation | 20.00 |
|  |  |  |  |  |  | Tree cover loss | 20.00 |
|  |  |  |  |  |  | Grassland loss | 20.00 |
|  |  |  |  |  |  | Wetland loss | 20.00 |
|  |  |  |  |  |  | Mineral depletion | 20.00 |
|  |  |  |  | Sanitation and water resources | 20.68 | Freshwater | 33.33 |
|  |  |  |  |  |  | Clean drinking water | 33.33 |
|  |  |  |  |  |  | Renewable internal freshwater resources | 33.33 |
|  |  |  |  | Hazardous chemicals | 17.52 | Fertilizer consumption | 14.28 |
|  |  |  |  |  |  | Controlled solid waste | 14.28 |
|  |  |  |  |  |  | So_2_ growth | 14.28 |
|  |  |  |  |  |  | Nox growth | 14.28 |
|  |  |  |  |  |  | Wastewater treatment | 14.28 |
|  |  |  |  |  |  | Electronic waste | 14.28 |
|  |  |  |  |  |  | Non-recycled municipal solid waste | 14.28 |
|  |  |  |  | Environmental biodiversity | 18.42 | Protected areas representativeness | 33.33 |
|  |  |  |  |  |  | Species habitat | 33.33 |
|  |  |  |  |  |  | Biodiversity habitat | 33.33 |
| Core drivers index (CDI) | 68.52 | Governance | 21.73 | Participation | 10.97 | Global connectivity | 25.00 |
|  |  |  |  |  |  | Risk communication | 25.00 |
|  |  |  |  |  |  | One Health association | 25.00 |
|  |  |  |  |  |  | One Health forums | 25.00 |
|  |  |  |  | Rule of law | 15.75 | General rule of law | 50.00 |
|  |  |  |  |  |  | One Health specialized law & regulation | 50.00 |
|  |  |  |  | Transparency | 9.98 | Transparency | 100.00 |
|  |  |  |  | Responsiveness | 12.56 | Emergency response operation | 50.00 |
|  |  |  |  |  |  | Exercising response plans | 50.00 |
|  |  |  |  | Consensus oriented | 10.84 | Consensus oriented | 50.00 |
|  |  |  |  |  |  | One Health education | 50.00 |
|  |  |  |  | Equity and inclusiveness | 13.79 | Zoonotic disease governance | 33.33 |
|  |  |  |  |  |  | Protected areas representativeness | 33.33 |
|  |  |  |  |  |  | Sustainable nitrogen management | 33.33 |
|  |  |  |  | Effectiveness and efficiency | 13.18 | Government effectiveness | 100.00 |
|  |  |  |  | Political support | 12.93 | One Health official department | 25.00 |
|  |  |  |  |  |  | Control of corruption | 25.00 |
|  |  |  |  |  |  | Regulatory quality | 25.00 |
|  |  |  |  |  |  | Government spending | 25.00 |
|  |  | Zoonotic diseases | 20.34 | Source of infection | 23.70 | Strategy and regulation | 41.32 |
|  |  |  |  |  |  | Surveillance and response | 33.36 |
|  |  |  |  |  |  | Sanitation | 25.32 |
|  |  |  |  | Route of transmission | 25.31 | Detection | 45.15 |
|  |  |  |  |  |  | Vector and reservoir interventions | 54.85 |
|  |  |  |  | Targeted population | 19.09 | Vaccination regulation | 28.98 |
|  |  |  |  |  |  | Population coverage and cost of interventions | 39.43 |
|  |  |  |  |  |  | Inhabitants below 5 meters above sea level | 31.60 |
|  |  |  |  | Capacity building | 16.77 | Zoonosis health promotion | 56.86 |
|  |  |  |  |  |  | Natural protected areas | 43.14 |
|  |  |  |  | Outcomes (case-studies) | 15.13 | COVID-19 confirmed cases | 25.42 |
|  |  |  |  |  |  | Human DALYs of echinococcosis | 15.84 |
|  |  |  |  |  |  | Human DALYs of leishmaniasis | 15.52 |
|  |  |  |  |  |  | Human DALYs of rabies | 20.33 |
|  |  |  |  |  |  | Human DALYs of tuberculosis | 22.88 |
|  |  | Food security | 21.35 | Food demand and supply | 20.00 | Food demand score | 21.76 |
|  |  |  |  |  |  | Food loss and waste | 20.20 |
|  |  |  |  |  |  | Infrastructures score | 19.42 |
|  |  |  |  |  |  | Food import score | 14.73 |
|  |  |  |  |  |  | Food production score | 23.89 |
|  |  |  |  | Food safety | 20.00 | Food safety governance | 30.33 |
|  |  |  |  |  |  | Food quality control | 26.68 |
|  |  |  |  |  |  | Food safety score | 22.41 |
|  |  |  |  |  |  | Foodborne illness burden | 20.58 |
|  |  |  |  | Nutrition | 20.00 | Food balance | 39.25 |
|  |  |  |  |  |  | Nutrition promoting capacity | 30.11 |
|  |  |  |  |  |  | Nutrition score | 30.64 |
|  |  |  |  | Natural and social circumstances | 20.00 | Famine warning | 22.55 |
|  |  |  |  |  |  | Natural sources sustainability in Land and Water | 24.89 |
|  |  |  |  |  |  | Economic performance index | 18.64 |
|  |  |  |  |  |  | Agriculture value added per worker | 18.24 |
|  |  |  |  |  |  | Food price indicators | 15.68 |
|  |  |  |  | Government support and response | 20.00 | Investment and financial support Score | 55.42 |
|  |  |  |  |  |  | Training and AI agriculture performance score | 44.58 |
|  |  | Antimicrobial resistance | 18.10 | Amr surveillance system | 20.00 | Antimicrobial consumption both in human and animals | 27.58 |
|  |  |  |  |  |  | Antimicrobial resistance status in human, animal and food | 41.63 |
|  |  |  |  |  |  | Environmental surveillance system | 30.79 |
|  |  |  |  | AMR laboratory network and coordination capacity | 20.00 | National AMR capacity | 37.06 |
|  |  |  |  |  |  | Technical promotion scores in AMR | 30.22 |
|  |  |  |  |  |  | National action plan formulations | 32.71 |
|  |  |  |  | Antimicrobial control and optimization | 20.00 | National law for antibiotic use | 38.33 |
|  |  |  |  |  |  | Optimization of antimicrobial use | 30.21 |
|  |  |  |  |  |  | Interruption capacity of antimicrobial resistance transmission | 31.46 |
|  |  |  |  | Improve awareness and understanding | 20.00 | Raising awareness and understanding | 50.00 |
|  |  |  |  |  |  | Professional training activities in multi-sectors | 50.00 |
|  |  |  |  | Antimicrobial resistance rate for important antibiotics | 20.00 | Carbapenems-resistance for multi-species, e.g., *Klebsiella pneumoniae, Acinetobacter baumannii, Escherichia coli, Pseudomonas aeruginosa* | 19.32 |
|  |  |  |  |  |  | Vancomycin-resistance for *Enterococcus faecium*, and *Enterococcus faecalis* | 15.11 |
|  |  |  |  |  |  | Third-generation Β-lactams-resistance for multi-species, e.g., *Staphylococcus aureus, Klebsiella pneumoniae, Escherichia coli, Pseudomonas aeruginosa* | 17.76 |
|  |  |  |  |  |  | Macrolides-resistance for Streptococcus pneumoniae | 14.01 |
|  |  |  |  |  |  | Aminoglycosides-resistance for *Klebsiella pneumoniae and Acinetobacter baumannii* | 16.51 |
|  |  |  |  |  |  | Quinolone-resistance for *Klebsiella pneumoniae, Escherichia coli, Acinetobacter baumannii* | 17.29 |
|  |  | Climate change | 18.48 | Government response | 37.91 | Climate policy | 38.82 |
|  |  |  |  |  |  | Climate knowledge system | 25.72 |
|  |  |  |  |  |  | Climate intervention strategy | 35.45 |
|  |  |  |  | Climate change risks | 29.59 | Air condition score | 26.05 |
|  |  |  |  |  |  | Extreme weather indicators, e.g. wildfires, droughts, floods, extreme temperatures | 21.86 |
|  |  |  |  |  |  | Energy use indicators, e.g. oil, natural gas, electricity, coal | 26.75 |
|  |  |  |  |  |  | Greenhouse gas emissions score | 25.35 |
|  |  |  |  | Health outcome | 32.50 | Directly health outcome indicators | 58.08 |
|  |  |  |  |  |  | Indirectly health outcome indicators | 41.92 |

**Note: Categories, key indicators and indicators are comparable to the first-level, second-level and third-level indicators presented in the main text.**

For each sub-indicator, we set the best/worst value for it, and use the following equation for normalization:

$$S_{ij}=\left\{ \frac{\begin{matrix} 0 \\ X_{ij}-X_{worst,j} \end{matrix}}{\begin{matrix} X_{best,j}-X_{worst,j} \\ 100 \end{matrix}}\times100 \right.$$

where $S_{\mathrm{ij}}$ denotes the normalized score for j-th sub-indicator of i-th country; $X_{\mathrm{ij}}$ denotes the original values for j-th sub-indicator of i-th country; $X_{best,j}$ denotes the original values of best performance for j-th sub-indicator; $X_{worst,j}$ denotes the original values of worst performance for j-th sub-indicator.

The weighted sum of the scores of the lower-level indicators (e.g., sub-indicators) was derived from the following equation to obtain the scores of the upper-level indicators (e.g., indicators):

$$S_{ih}=\sum_{1_{h}}^{m_{h}} S_{\mathrm{ij}_{h}}\times W_{j_{h}} , \sum_{1_{h}}^{m_{h}} W_{j_{h}}=1$$

where $S_{ih}$ denotes the score of the h-th upper-level indicator of i-th country; $m$ denotes the total number of the lower-level indicators under h-th upper-level indicator; $j_{h}$ denotes the j-th lower-level indicator under h-th upper-level indicator; $S_{\mathrm{ij}_{h}}$ denotes the score of$j_{h}$-th lower-level indicator of i-th country; $W_{j_{h}}$ denotes the weight of $j_{h}$-th lower-level indicator.
